# Book Reviews

**DOI:** 10.1155/S1463924679000468

**Published:** 1979


### Book Reviews

### Managing Management Information Systems

**by Phillip Ein Dor and Eli Seger**

*Lexington Books, 1978, 191pp, £12.00 USBN 0669 01642-x*

In the preface to this book the authors describe the development of management information systems as an art not a science. From many of their statements it is abundantly clear that they see the situation with respect to designers and users of MIS in much the same way that exists in the field of automation itself. A management information system is a system (a data base) which a manager interrogates or wishes to interrogate to provide valuable information, which will aid his judgement in management situations. It is a natural development of automatic analytical systems to generate a large data base of information as it is natural for a systems designer working in laboratory automation to be aware of the economic connotations of his work these facts should stimulate an interest in the management of information systems. This book therefore, will provide a valuable backcloth for discussion and will prompt the reader into questioning some of his own views on the subject.

It is very often taken for granted that the user requirements for an MIS can be accurately specified in advance. However, the authors clearly question this because managers in their experience feel threatened by the information systems development and by the 'prying' of the analyst into their former preserves. When this latter situation exists there is a tendency to make omissions. to exaggerate or be inaccurate, vague or non specific.

The various ways around this problem are described in many parts of the book but in addition to the managers' fears there is the additional problem that the systems designers shroud themselves in jargon and form an independent subculture of their own. The managers confronted with this tend to be either intimidated or angered, especially if it appears that excuses are being continually made as to why he cannot have what he asks for. If this book does no more than show up this area of communications difficulty and expose it to both the user and the implementor it will be very worth its value. To the senior management who perhaps initiate the needs for such information systems it will point out to be tactful and to ensure that the initial specification and implement stages are carried out effectively. Rushing into a system implementation may provide some initial advantages but these may soon be lost if the system cannot grow with the organisation and be modified to take account of damaging needs. The requirement to provide the necessary education to all levels of management and designers is clearly set out in this book.

The book is clearly presented and various chapters deal with the various facets of the problem. In the main it concentrates in two subjects, procedural aspects and human aspects. The authors conclusions are drawn together at the close of the book, these are extremely constructive and should help many of us to improve the efficiency of our management information systems. Very often managers complain that their particular system does not give them what they need, it is often the case that their needs as they now define them were never specified at the design stage. In short a good example of 'garbage in' giving 'garbage out'. A short study of this book may help them overcome this problem. For many systems it will be too late but for others it could be the key to success.

*P.B. Stockwell*

### Automation and Mechanization of Column Operations in Liquid Chromatography

**by B. Meloun**, in *Laboratory Handbook of Chromatographic and Allied Methods*, Edited by O. Mikes and translated from the Czech by Z. Prochazka.

*1979, Ellis Horwood, Chichester, pp 764, £38.50. ISBN 0 85312-080-3*

To users of currently available liquid chromatography equipment the title of this chapter might be seen as a misnomer, though this might be attributable to the translation. The chapter deals more with the mechanisms and equipment used in column chromatography rather than what is normally meant by automation and mechanization. The author starts by describing a block diagram which illustrates the functional parts of a chromatographic system, starting with solvent reservoir going on to pump, injection system, column, detector and fraction collector. Each of these functional blocks is then discussed in some detail. The subject of gradient elution and associated programming devices is treated in considerable detail and several pages are devoted to detectors. There are numerous diagrams which are variable in execution, those for simple objects such as pipe fittings, and septum injector ports are almost of the quality of engineering drawings whilst more complex items are treated much more simply. Towards the end of the chapter a section on recording and calculation and one on complex systems go some way to justifying the automation in the title, as the automatic analysis of amino acids is used to demonstrate the functions of an integrator, and a Technicon AutoAnalyzer system is used to perform post-column determinations of two enzymes and total protein. This latter system could have been dealt with in more detail and would thus have been more useful as an example of this technique.

The chapter concludes with a list of about thirty manufacturing companies mentioned in the text and a bibliography of forty-nine references.

*T.G. Alliston*

**The following papers are expected to be published in forthcoming issues of *The Journal of Automatic Chemistry*.**

A microprocessor controlled liquid chromatograph/atomic absorption system.
*by T.M. Vickrey and W. Eue.*

Assessment of the ENI Gemini microprocessor controlled centrifugal analyser.
*by N. Potezney, R.G. White and T.D. Geary.*

Microcomputer automated recording spectropolarimeter.
*by V.C. Zadnik, J.L. Scott, R, Megargle, J. Kerkay and K.H. Pearson.*

Design considerations involved in the development of an automatic hydride evolution system for the measurement of nanogram amounts of arsenic, selenium and antimony by atomic spectroscopy.
*by D.G. Porter and A.L. Dennis.*

Continuous monitoring using polarographic electrodes.
*by L.C. Clark.*

Automatic system for quality control of dyestuffs: design philosophy and implementation.
*by H.C. Metz and G. Michel.*

